# Mitochondrial Ca^2+^-Handling in Fast Skeletal Muscle Fibers from Wild Type and Calsequestrin-Null Mice

**DOI:** 10.1371/journal.pone.0074919

**Published:** 2013-10-03

**Authors:** Michele Scorzeto, Marta Giacomello, Luana Toniolo, Marta Canato, Bert Blaauw, Cecilia Paolini, Feliciano Protasi, Carlo Reggiani, Ger J. M. Stienen

**Affiliations:** 1 Department of Biomedical Sciences and Interuniversity Institute of Myology (IIM), University of Padova, Padua, Italy; 2 Venetian Institute of Molecular Medicine, Padua, Italy; 3 Department of Neuroscience and Imaging (DNI) and Center for Research on Ageing (CeSI), and Interuniversity Institute of Myology (IIM), University G. d' Annunzio, Chieti, Italy; 4 Laboratory for Physiology, Institute for Cardiovascular Research, VU University Medical Center, Amsterdam, The Netherlands; 5 Department of Physics and Astronomy, VU University Amsterdam, Amsterdam, The Netherlands; Tohoku University, Japan

## Abstract

Mitochondrial calcium handling and its relation with calcium released from sarcoplasmic reticulum (SR) in muscle tissue are subject of lively debate. In this study we aimed to clarify how the SR determines mitochondrial calcium handling using dCASQ-null mice which lack both isoforms of the major Ca^2+^-binding protein inside SR, calsequestrin. Mitochondrial free Ca^2+^-concentration ([Ca^2+^]_mito_) was determined by means of a genetically targeted ratiometric FRET-based probe. Electron microscopy revealed a highly significant increase in intermyofibrillar mitochondria (+55%) and augmented coupling (+12%) between Ca^2+^ release units of the SR and mitochondria in dCASQ-null vs. WT fibers. Significant differences in the baseline [Ca^2+^]_mito_ were observed between quiescent WT and dCASQ-null fibers, but not in the resting cytosolic Ca^2+^ concentration. The rise in [Ca^2+^]_mito_ during electrical stimulation occurred in 20−30 ms, while the decline during and after stimulation was governed by 4 rate constants of approximately 40, 1.6, 0.2 and 0.03 s^−1^. Accordingly, frequency-dependent increase in [Ca^2+^]_mito_ occurred during sustained contractions. In dCASQ-null fibers the increases in [Ca^2+^]_mito_ were less pronounced than in WT fibers and even lower when extracellular calcium was removed. The amplitude and duration of [Ca^2+^]_mito_ transients were increased by inhibition of mitochondrial Na^+^/Ca^2+^ exchanger (mNCX). These results provide direct evidence for fast Ca^2+^ accumulation inside the mitochondria, involvement of the mNCX in mitochondrial Ca^2+^-handling and a dependence of mitochondrial Ca^2+^-handling on intracellular (SR) and external Ca^2+^ stores in fast skeletal muscle fibers. dCASQ-null mice represent a model for malignant hyperthermia. The differences in structure and in mitochondrial function observed relative to WT may represent compensatory mechanisms for the disease-related reduction of calcium storage capacity of the SR and/or SR Ca^2+^-leakage.

## Introduction

In all excitable and non-excitable cells, mitochondria take up and release Ca^2+^ in a well regulated manner. This Ca^2+^ transport is crucial for the regulation of mitochondrial activity [1] and is involved in the cascade of events leading to apoptosis [2], [3]. It is well established that the activity of a number of key metabolic enzymes and the F0-F1 ATPase (ATP synthase) itself is controlled by the Ca^2+^ concentration inside the mitochondrial matrix ([Ca^2+^]_mito_) [4], [1]. This regulation is of special (patho)physiological interest in muscle tissue, where the high energy cost of contractile activity requires large (even 100 fold) and fast variations of aerobic ATP production [5], [6].

Ca^2+^ transport through the mitochondrial membranes has been the object of several studies in the last ten years, which have demonstrated that mitochondrial free Ca^2+^ concentration undergoes rapid variations in parallel with the cytosolic Ca^2+^ transients [7−10]. The low affinity mitochondrial Ca^2+^ uniporter (MCU) which has recently been identified as a 40-kDa protein located in the inner mitochondrial membrane [11], [12] and the mitochondrial Na^+^/Ca^2+^ exchanger (mNCX), e.g. [13] are considered as major pathways for mitochondrial Ca^2+^ uptake and extrusion, respectively.

Evidence has been presented that microdomains are present near the Ryanodine receptor (RyR) where the local Ca^2+^ concentration is high during the initial phase of the SR Ca^2+^-release process, see for a review [14], [15]. Accordingly, the close proximity of mitochondria to the sarcoplasmic reticulum (SR), strengthened by the presence of small (10 nm) electron-dense linkages, or tethers [10], [16], would facilitate exchange of Ca^2+^ between the mitochondria and the SR during contraction and relaxation in skeletal muscle.

Our previous studies [17−19] have shown that genetic ablation of calsequestrin has a very important effect on intracellular calcium homeostasis detectable when either the fast isoform CASQ1 or both CASQ1 and CASQ2 (the slow isoform) are removed. In those conditions Ca^2+^ available inside the SR becomes insufficient for sustained contractile activity and muscle fibers become dependent on extracellular Ca^2+^ which might enter the cells via store-operated Ca^2+^ channels (SOCE) [17)]. Indeed, increased SOCE has been reported in skeletal muscle with reduced calsequestrin-1 expression [20]. In addition, the ablation of calsequestrin has been shown to be accompanied with an increase in mitochonchondrial volume [21] which might represent an additional compensatory mechanism. Therefore, the aim of this study was to determine whether and to which extent mitochondrial Ca^2+^ handling is affected by a depletion of the SR calcium store and by the lowering of extracellular Ca^2+^ concentration.

The intramitochondrial free Ca^2+^ concentration [Ca^2+^]_mito_ was studied in adult skeletal muscle fibers using a ratiometric FRET-based probe (4mtD3cpv) which is specifically targeted to the mitochondrial matrix [22]. A comparison was made between the free mitochondrial Ca^2+^ concentration in dCASQ-null (lacking both CASQ isoforms) and wild type fibers both at rest and during contractile activity induced by electrical stimulation. In addition we used electron microscopy to study the differences in mitochondrial density and in the local arrangement of the mitochondria and the SR between the dCASQ-null and wild type fibers.

The mitochondrial free Ca^2+^ concentration was found to be higher at rest but lower during contraction in dCASQ-null fibers compared to wild type fibers. Detailed analysis of the kinetics of the changes in mitochondrial free Ca^2+^ concentration during contraction revealed significant differences between dCASQ-null and WT fibers and significant changes therein upon removal of extracellular Ca^2+^. This indicates that mitochondrial Ca^2+^-handling in fast skeletal muscle fibers is sensitive to the calcium available in both intracellular and extracellular Ca^2+^-stores. The importance of these findings for altered muscle function in malignant hyperthermia will be discussed.

## Materials and Methods

### Animals

Experiments were carried out on 4 months old dCASQ-null and wild type (WT) mice. Double (d)CASQ-null mice [23] were generated by crossing CASQ1-null mice [18] with CASQ2-null mice [24]. Wild type C57BL/6J mice, same strain as dCASQ-null mice, were purchased from Charles River Laboratories (Wilmington, MA, USA). The use of the animals and the experimental protocol was approved by the Ethical Committee and by the animal welfare coordinator of the University of Padua. Mice were maintained in an accredited animal house and examined daily.

### Transfection

4mtD3cpv cameleon targeted to the mitochondrial matrix via a targeting sequence, derived from subunit VIII of human cytochrome *c* oxidase (COX), and replicated 4 times [25] in pcDNA3 (kindly donated by R.Y. Tsien (University of California, San Diego, CA, USA)) was used in transfection experiments. The structural and functional characterization of 4mtD3cpv is reported by Palmer et al. [22]. FDB muscles were transfected in vivo as described previously [26].

### Isolation and culture of adult muscle fibers

Enzymatic isolation of single skeletal muscle fibers from FDB muscle was performed using collagenase treatment [23]. Isolated cells were seeded on laminin coated coverslips and left to adhere and to stabilize overnight in tissue culture medium at 36°C. The next morning the coverslips were mounted in a measuring chamber containing imaging buffer (composition in mM: NaCl 125, KCl 5, CaCl_2_ 1, MgSO_4_ 1, KH_2_PO_4_ 1, glucose 5, HEPES 20, pH 7.2) and equipped with platinum field electrodes. The zero external calcium solution used in part of the experiments had an identical composition except that CaCl_2_ was omitted and 50 µM EGTA was added to scavenge any remaining Ca^2+^-ions. Fibers were incubated for 1 hour, prior to the measurements. As in our previous experiments we did not add MgCl_2_ to replace CaCl_2_. Temperature during the measurements was kept at 25−26°C, in order to slow the kinetics of Ca^2+^ release and reuptake into the mitochondria, and to facilitate comparison with previous studies [17], [18], [21].

### Immunocytochemistry

Fibers were fixed with 4% formalin in PBS for 30 minutes, permeabilized with 1% Triton and blocked in 10% goat serum for 1 hour to avoid non-specific signal. Fibers were incubated overnight at 4°C with primary antibodies specific for mitochondrial outer membrane protein Tom20 (FL-145, SantaCruz Biotechnology, Santa Cruz, USA) and ryanodine receptor (R129, Sigma-Aldrich, St. Louis, MO, USA) and with appropriate fluorescent secondary antibodies for 2 h at room temperature. The fibers were viewed with a confocal microscope (Leica SP2, Leica Microsystems, Rijswijk, Netherlands).

### Electron microscopy (EM)

Electron-micrographs were obtained from FDB muscles as described previously [17], [21]. Intermyofibrillar and subsarcolemmal mitochondrial density and their position relative to the CRUs were determined from 10 micrographs for each fiber (at 11,000X) of non-overlapping regions that were randomly collected from longitudinal sections. 10 fibers were analyzed in each animal (2 WT and 2 dCASQ-null mice) in a blinded fashion. Mitochondria and CRU/mitochondria couples were marked and counted in each micrograph and the area of the image was determined. Mitochondrial volume was determined using the well-established stereology point-counting techniques [27], [28] in EM images taken at 14,000X of magnification after superimposing an orthogonal array of dots at a spacing of 0.20 μm to the electron micrographs. Subsarcolemmal mitochondria were counted in a layer underneath the sarcolemma with a thickness of 1 μm.

### Measurements of the intramitochondrial free Ca^2+^-concentration

Measurements of the intramitochondrial free Ca^2+^-concentration ([Ca^2+^]_mito_) were carried out using an inverted fluorescence microscope (Eclipse-Ti, Nikon Instruments, Amsterdam, Netherlands) at 20X magnification equipped with the perfect focus system (Nikon Instruments) and a cooled CCD camera (C9100-13, Hamamatsu), essentially as described previously [17]. A relatively high excitation intensity of the YFP and CFP fluorophores and an image size of 17×112 pixels per channel was required to achieve high time resolution (8 ms) in the measurements. The effect of bleaching of the Ca^2+^-indicator was studied in fibers stimulated at 0.1 Hz and occasionally at 0.033 Hz. At such low frequency of stimulation the recovery in the YFP/CFP ratio after the contraction is almost complete before the next stimulus starts and fibers, in addition, do not fatigue during the measurements. The results and the method to correct for bleaching are shown in the Supporting Information (**Fig. S1 in File S1**).

In a separate set of experiments, the time resolution of the recordings was reduced to 900 ms by increasing the integration time of the CCD camera and reducing the illumination intensity. In this way bleaching of the probe was negligible and the duration of the recordings could be increased to assess very slow changes in Ca^2+^ inside the mitochondria. In these latter experiments the effect of the mitochondrial Na^+^/Ca^2+^ exchanger blocker (CGP37157, 1 μM; Tocris, Bristol, UK) was assessed.

### Data analysis

The correction for bleaching and the subsequent analysis of the recordings was performed using a computer program written in MatLab (MatWorks, Natick, MA, USA). A third order polynomial fit to the initial and final 5-s periods of the recordings was calculated and subsequently subtracted from the data points to correct for the change in baseline as a result of bleaching. The intercept at t = 0 s of the polynomial fit to the baseline of the first fiber recorded in each dish was used to estimate the basal level of the free mitochondrial Ca^2+^-concentration in quiescent fibers. Additional information on the correction for bleaching of the probe is given in the Supporting Information (**Fig. S2 in File S1**)

The time averaged steady-state responses during a train of stimuli at 1 Hz were used for the analysis of the early rapid alterations in free [Ca^2+^]_mito_. The rise time (t_10–90%_) of the increase in free [Ca^2+^]_mito_ from 10 to 90% of the peak amplitude was calculated from the interpolated recording after correction for bleaching. The decline in free [Ca^2+^]_mito_ during 1 Hz stimulation was fitted with a double exponential: y(t) = a_0_+a_1_exp (−k_1_⋅t) + a_2_exp (−k_2_⋅t). The value of this equation at t = 1 second was used as the minimum value of the averaged recording in the determination of the rise time in order to minimize the effect of noise. The amplitude a_1_ was calculated by extrapolating the double exponential to t = 0 seconds, the moment at which the maximum value was reached. The decline in free [Ca^2+^]_mito_ after the train of stimuli was fitted to a single exponential: y(t) = a_3_⋅exp (−k_3_⋅t), in which the parameter a_3_ reflected the decrease in free [Ca^2+^]_mito_ after the train of stimuli as well as the (quasi steady-state) increase in free [Ca^2+^]_mito_ during the train of stimuli.

To resolve very slow components of the decline in free [Ca^2+^]_mito_, we extended the recording of the decay phase after a train of 5 Hz stimulation to 10 minutes. In these recordings the integration time of the camera was increased to 900 ms and the illumination intensity was reduced accordingly in order to reduce bleaching of the probe. The final decline in free [Ca^2+^]_mito_ in these recordings was fitted to a single exponential: y(t) = a_4_⋅exp (−k_4_⋅t).

### Calibration of the cameleon (4mtD3cpv)

Calibration of the Ca^2+^ sensor was performed as describes previously [29]. The equation, [Ca^2+^]  = β·K'_d_·[(R-R_min_)/(R_max_-R)]^1/n^ was used to estimate the free [Ca^2+^]_mito_ in quiescent fibers as well as the levels reached at the end of stimulation, using a K'd of the 4mtD3cpv sensor of 0.76 μM and n value of 0.74 [22]. Details on the estimation of Rmin and Rmax are given in File S1. A value of β of 6 was adopted to account for the difference between the *in vitro* and *in situ* K'd value [30].

### Cytosolic Ca^2+^ determination with Fura-2 AM

Fibers were loaded with 5 µM Fura-2 acetoxymethyl ester (Life Technologies, Bleiswijk, Netherlands) in incubation buffer as described previously [18]. After a minimum of 30 minutes, Ca^2+^ signals were recorded using a dual-beam excitation fluorescence photometry setup (IonOptix Corp. Milton, MA, USA) at 25–26°C. Calibration of the Fura-2 signals and calculation of the free cytosolic Ca^2+^ concentration were as reported previously [17].

### Statistical analysis

Data are expressed as mean ± SEM. Comparisons between groups were made using two-way ANOVA (Type x Ca^2+^ or Ca^2+^ x Stimulation Frequency) or three-way ANOVA (Type x Ca^2+^ x Stimulation Frequency (repeated measures), respectively) followed by Bonferroni post hoc test, if appropriate, to assess differences between WT vs. dCASQ-null, 1 mM vs. 0 external Ca^2+^, at the various stimulation frequencies used. P<0.05 was considered statistically significant.

## Results

### Intracellular distribution of the probe in enzymatically dissociated FDB muscle fibers

**Figure 1:**
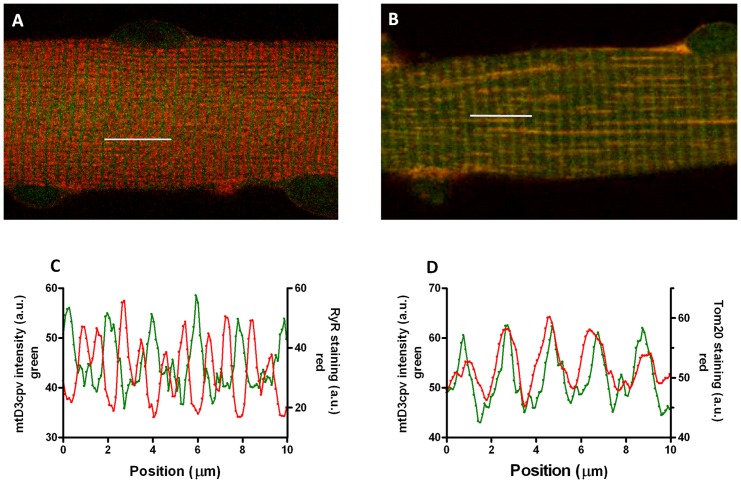
Localization of 4mtD3cpv in FDB muscle fibers. **A** and **C**: mitochondrial cameleon (4mtD3cpv) fluorescence (green) and anti-RyR antibody staining (red) in a merged image (A) and intensity profiles (C). **B** and **D**: mitochondrial cameleon (4mtD3cpv) fluorescence (green) and anti-Tom20 antibody staining (red) in a merged image (B) and intensity profiles (D). Segments 10 μm long were scanned on the images as indicated by the white bars.

**Figure 2:**
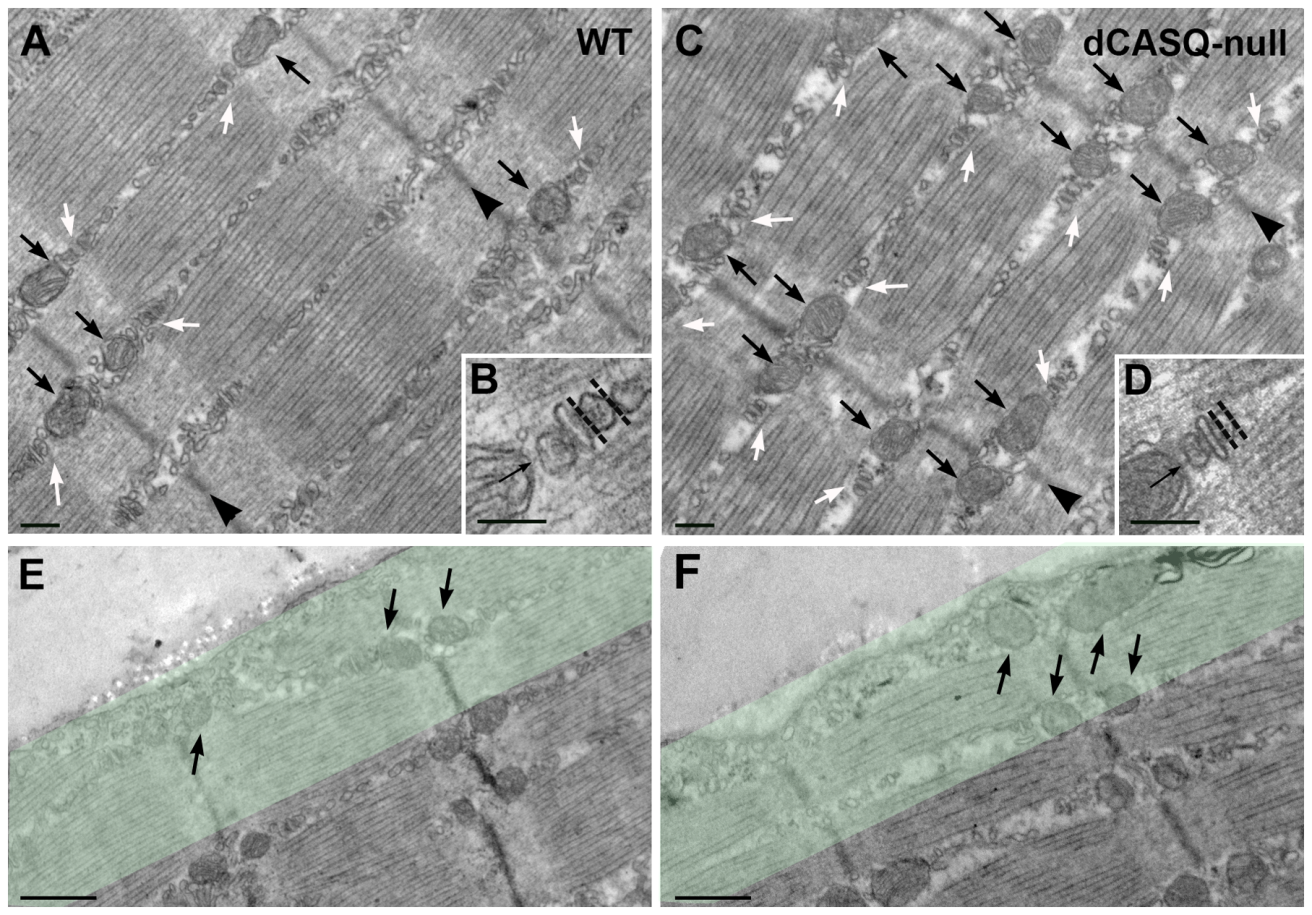
Structural changes in mitochondria and CRUs. **A** and **C.** The intermyofibrillar mitochondria (small black arrows) are predominantly located at the I-band, next to CRUs or triads (white arrows) on both sides of Z-lines (arrowheads). **B.** and **D.** Enlargements of CRUs next to intermyofibrillar mitochondria. The two organelles are tethered together by small electron-dense bridges (arrows), whereas the width (dotted lines) of the terminal cystenae is reduced in dCASQ-null relative to WT. **E.** and **F.** Subsarcolemmal mitochondria (arrows) in WT and dCASQ-null, respectively. The green band indicates the 1 μm-wide region where frequency and volume fraction of subsarcolemmal mitochondria were determined. Scale bars, A and C: 0.2 μm; B and D: 0.1 μm; E and F: 0.5 μm.

4mtD3cpv transfected fibers stained with an antibody specific for the mitochondrial protein Tom20 and an antibody specific for the ryanodine receptor (RyR) showed a striated sarcomeric pattern (**Fig. 1**). Colocalization of 4mtD3cpv and the mitochondria was confirmed in the merged image and in the superimposed intensity profiles (**Fig. 1B and D**). Antibody staining for RyR and 4mtD3cpv showed a “sandwich” pattern (**Fig. 1A and C**) as expected on the basis of the position of the Ca^2+^ release units relative to the mitochondria, resolved by electron microscopy (EM, see **Fig. 2**).

### Structural changes in mitochondria and their coupling with Ca^2+^ release units

The EM images shown in **Fig. 2** illustrate that myofibrillar morphology in dCASQ-null fibers was well maintained. However, quantitative analysis revealed that intermyofibrillar mitochondrial density was increased in dCASQ-null (n = 195 micrographs) relative to WT fibers (n = 194) from 45.0±2.6 to 69.7±2.1 per 100 μm^2^ i.e. by 55±10%. The percentage of mitochondria coupled to Ca^2+^ release units (CRUs) was already high in WT fibers (70.4±0.8%) and showed a further significant increase (+12.0±0.2% P<0.0001) up to 78.9±0.7% in dCASQ-null. In addition, the width of the terminal cisternae of the SR in dCASQ-null fibers was reduced (Fig.****2B, D, dotted line) relative to WT, c.f. [18], [21]. The intermyofibrillar mitochondrial volume fraction was increased from 4.3±0.2% in WT (n = 183) to 7.2±0.2% in dCASQ-null fibers (n = 192), i.e. by 67±6%. This increase corresponds well to the increase in mitochondrial density, indicating that intermyofibrillar mitochondrial shape and surface to volume ratio in WT and dCASQ-null are similar.

The subsarcolemmal mitochondrial volume was rather similar in WT and dCASQ-null and amounted to 5.8±0.5% in WT (n = 120) and 4.7±0.5% in dCASQ-null (n = 157). Subsarcolemmal mitochondria were counted in a layer of 1 μm in width and the typical radius of the fibers studied was 20 μm. The subsarcolemmal volume fraction thus represents 1-(19/20)^2^ or 10% of the total volume. This indicates that the intermyofibrillar mitochondrial volume fractions and the differences therein between WT and dCASQ-null also apply to the overall mitochondrial volume fractions in WT and dCASQ-null fibers.

### Free [Ca^2+^] in the mitochondrial matrix in quiescent fibers

**Figure 3:**
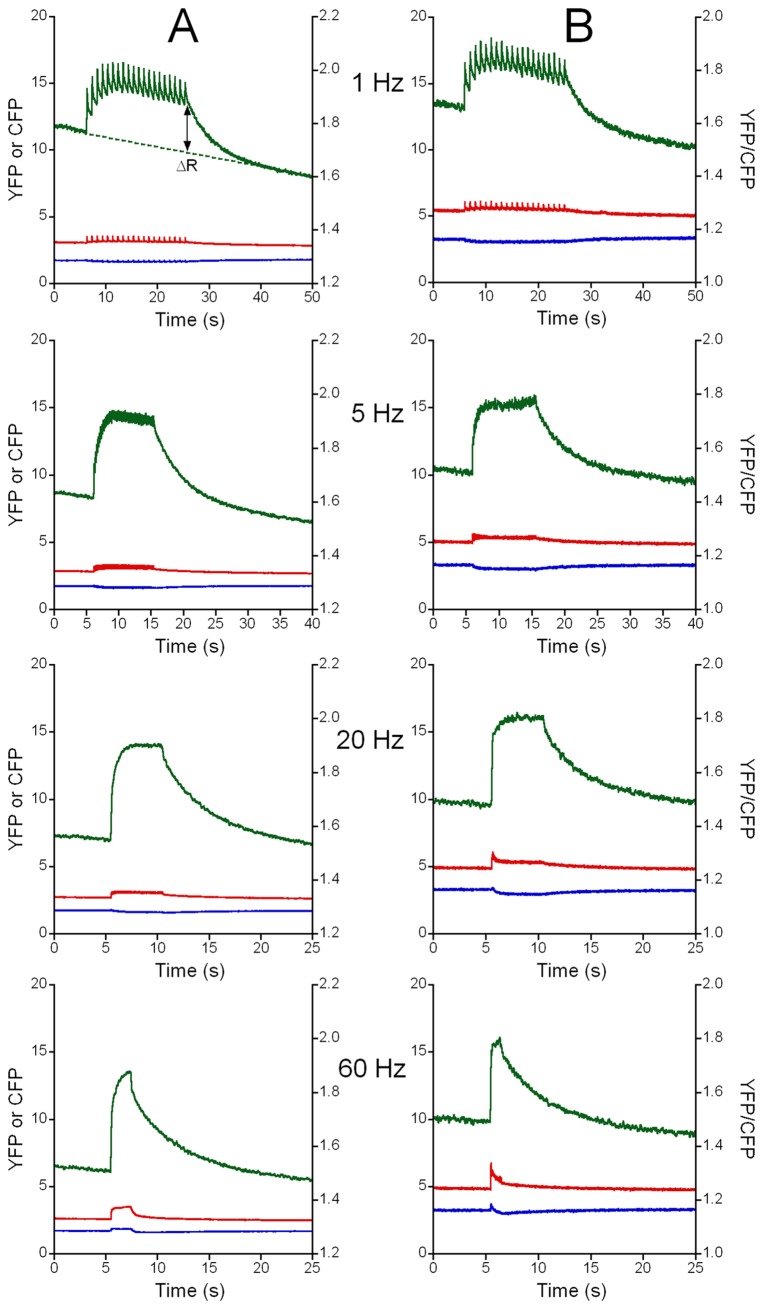
Changes in Ca^2+^ concentration in the mitochondrial matrix ([Ca^2+^]_mito_) during repetitive stimulation. The 4mtD3cpv responses (YFP, red; CFP, blue; YFP/CFP ratio, green) are shown in a WT fiber (**A**) and in dCASQ-null fiber (**B**) at 1 Hz, 5 Hz, 20 Hz and 60 Hz in the presence of 1 mM extracellular Ca^2+^. The YFP/CFP ratio is a measure of the free [Ca^2+^]_mito_. During stimulation at 1 Hz, the pulsatile increase in free [Ca^2+^]_mito_ during each contraction can be identified; at higher stimulation frequencies the free [Ca^2+^]_mito_ increases more smoothly during the train of stimuli. After the train of stimuli the [Ca^2+^]_mito_ signal returned to baseline (indicated by the dotted line in the WT recording at 1 Hz). The increase in the YFP/CFP ratio relative to the baseline (ΔR), indicated by the double sided arrow, is a measure of the increase in [Ca^2+^]_mito_ during the train of stimuli. In the YFP signal, rapid increases in intensity can be observed, partly caused by repetitive contractions of the fiber (see File S1).

Free [Ca^2+^]_mito_ was assessed from the ratio (R) of the YFP and CFP intensities in single WT and dCASQ-null fibers both in the presence and absence of external Ca^2+^. The initial recordings from each fiber were used to determine the basal values of the YFP/CFP ratios in order to eliminate any possible effect of electrical stimulation and bleaching of the Ca^2+^-sensor. The basal values were calculated from the intercept at time  = 0 of the polynomial fit of the baseline (dotted line in **Fig. 3**). The average basal values of R amounted to 1.84±0.02, 1.79±0.05, 1.93±0.03 and 1.96±0.05 in WT + Ca (n = 74), WT-Ca (n = 22), dCASQ-null + Ca (n = 39) and dCASQ-null-Ca (n = 15) fibers, respectively. ANOVA indicated a highly significant increase in free [Ca^2+^]_mito_ in dCASQ-null fibers relative to WT fibers (P<0.001), whereas no significant differences were observed between the measurements in the presence and absence of external Ca^2+^. The average values of the pooled data amounted to 1.83±0.02 in WT fibers (n = 96) and to 1.94±0.03 in dCASQ-null fibers (n = 54).

### Accumulation of mitochondrial Ca^2+^ during contractions induced by electrical stimulation

Representative examples of the recordings obtained during contractions elicited by trains of electrical stimulation at 1, 5, 20 and 60 Hz in fibers from WT and dCASQ-null animals are shown in **Fig. 3**. An example of the increase in mitochondrial calcium concentration upon a single electrical pulse (a twitch response) is shown in **Fig. S3 in File S1**. The general patterns in the recordings in WT and dCASQ-null fibers are rather similar. Periodic increases in YFP/CFP ratio, and thus in free [Ca^2+^]_mito_ were observed during repetitive electrical stimulation at 1 Hz. The rising and decay phases of the mitochondrial Ca^2+^ transients were highly asymmetric and this resulted in incomplete recovery of free [Ca^2+^]_mito_ during the contractions even at low stimulation frequency. During the first contractions, the transients were accompanied by a gradual increase relative to the baseline, reflecting an accumulation of Ca^2+^ inside the mitochondrial matrix, reaching a dynamic steady state that was maintained during the remainder of the train of stimuli. At the stimulation rate of 5 Hz mitochondrial Ca^2+^ accumulated more rapidly and [Ca^2+^]_mito_ reached a higher final level than in those at 1 Hz, while during the recordings at 20 and 60 Hz the rates and the levels attained were only slightly higher than those at 5 Hz. Whereas in WT fibers the accumulation of calcium in mitochondria continued during the stimulation period even when cytosolic calcium concentration was stable and constant, in dCASQ-null fibers, the initial fast rise in [Ca^2+^]_mito_ was followed by a slow increase or, in many instances, by a clear interruption of calcium accumulation, see for example the 60 Hz recording shown in **Fig. S4 in File S1**. This behavior may be linked to the decline in cytosolic free [Ca^2+^] and in contraction strength observed previously (17,21) in CASQ1-null and dCASQ-null fibers and suggests that the decline in cytosolic free [Ca^2+^] limited further increase in [Ca^2+^]_mito_ in dCASQ-null fibers.

**Figure 4:**
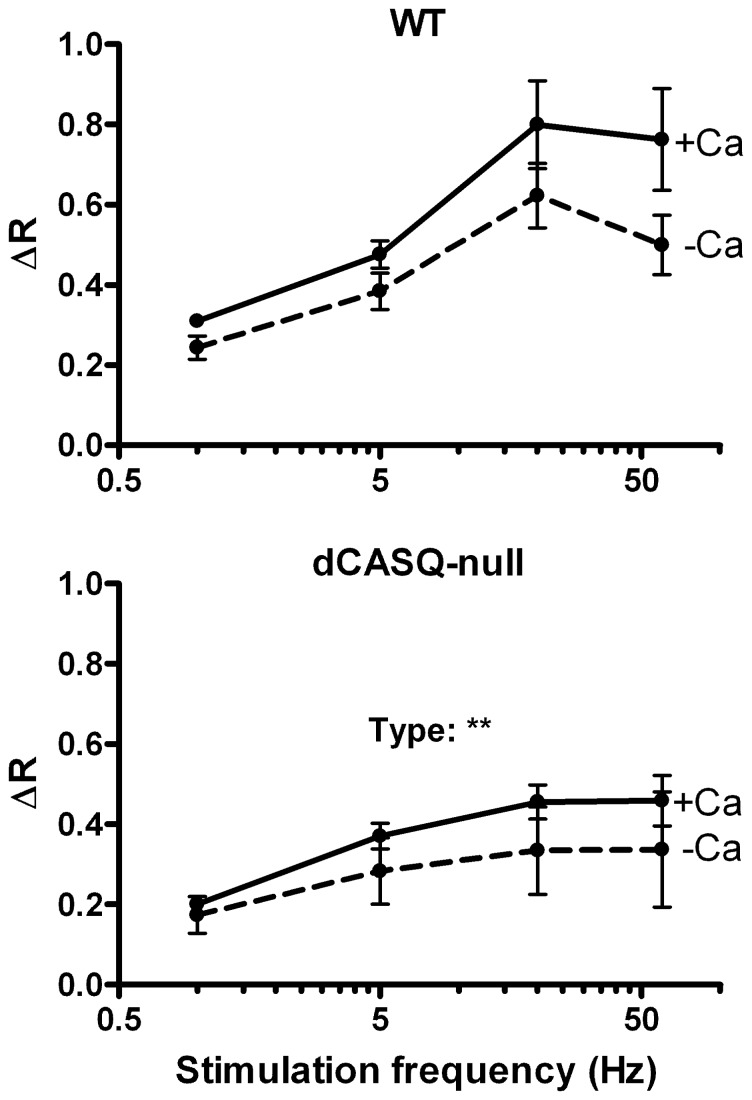
Average amplitudes of the increase in the YFP/CFP ratio (ΔR) during contractile activity. In the presence of 1^2+^, ΔR (after correction for bleaching) increased with stimulation frequency in the range from 1–20 Hz in both WT and dCASQ-null fibers. The increases in [Ca^2+^]_mito_ at 60 Hz were very similar to those at 20 Hz and the values reached were significantly smaller in dCASQ-null fibers than in WT fibers (** P<0.01). The averaged values in the absence of extracellular Ca^2+^ (-Ca) are smaller than the corresponding values in the presence of Ca^2+^, but the difference was not statistically significant (3-way ANOVA).

The overall mitochondrial Ca^2+^ accumulation during the train of stimuli was quantified by measuring the drop in the YFP/CFP ratio at the end of the train of stimuli (ΔR), after correction for bleaching of the probe as described in the Supporting Information (**Fig. S2 in File S1**). The averaged data obtained in WT and dCASQ-null fibers are shown in **Fig. 4**. The increases in free [Ca^2+^]_mito_ in dCASQ-null fibers were significantly smaller than in WT fibers (P<0.01) and somewhat blunted in the absence of extracellular Ca^2+^.

### Kinetics of mitochondrial calcium accumulation

**Figure 5:**
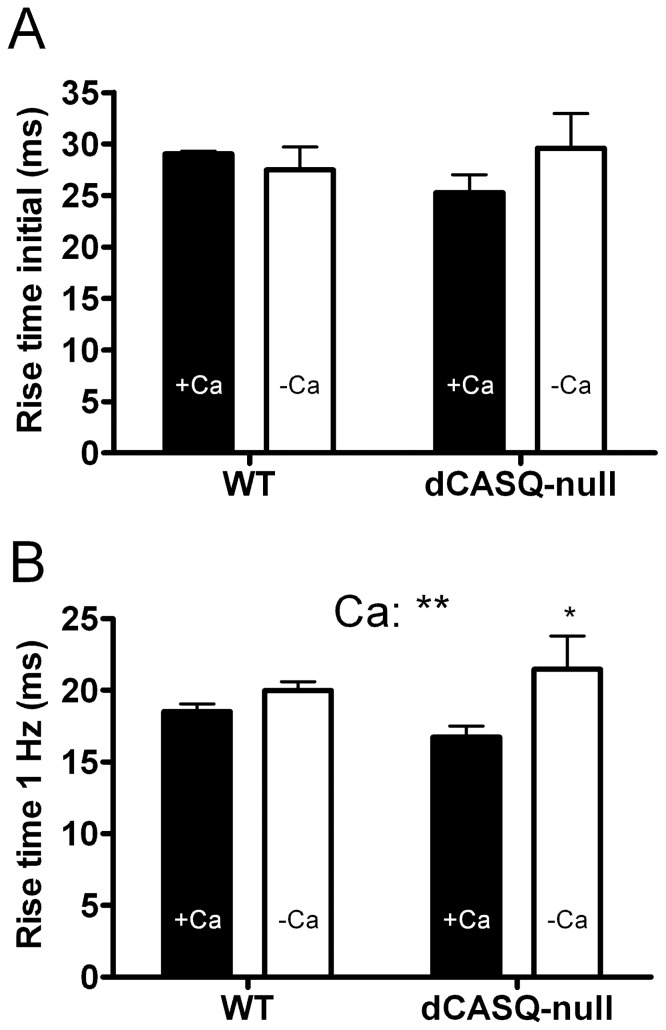
Rise time of the increase in [Ca^2+^]_mito_. **A.** Rise time (10–90%) of the first responses after quiescence at 0.1 and 1 Hz stimulation. No significant differences were observed between groups. **B.** Rise time (10–90%) obtained from the time averaged steady state response during 1 Hz stimulation. ANOVA indicated a very significant difference between the values obtained in the presence (filled bars) and absence (open bars) of extracellular Ca^2+^ (P<0.01), in particular in the dCASQ-null group (P<0.05). Note that the overall values in A (∼28 ms) are larger than the values in B (∼18 ms). Number of fibers: WT + Ca: 50; WT-Ca: 18; dCASQ-null + Ca: 22; dCASQ-null-Ca: 4. ** and * denote, respectively, P<0.01 and P<0.05 in a Bonferroni post hoc test.

Information on the kinetics of the rising phase of the mitochondrial calcium transient was obtained in twitches or the first contractions during stimulation trains at 0.1 and 1 Hz (**Fig. 5A**). These contractions were all initiated in rested fibers. No significant differences were observed between groups and the average rise time from 10% to 90% of the increase in [Ca^2+^]_mito_ (t_10–90%_) for the pooled data amounted to 27.8±1.6 ms (n = 179).

The analysis of averaged responses at 1 Hz allowed us to estimate t_10–90%_ during repetitive 1 Hz stimulation, when the transient responses had reached a steady state. The average t_10–90%_ from all groups amounted to 18.5±0.4 ms (n = 94). This value is significantly smaller (−33%) than the value obtained during the initial response in rested fibers.

There were no significant differences between WT and dCASQ-null but small, highly significant differences (P<0.01, ANOVA) were observed in the presence and absence of external Ca^2+^ (**Fig. 5B**). Posthoc analysis indicated a significant prolongation of rise time in dCASQ-null fibers from 16.7±0.8 ms in the presence (n = 22) to 21.5±2.3 ms in the absence (n = 4) of external Ca^2+^ (P<0.05).

### Kinetics of mitochondrial [Ca^2+^] decay

**Figure 6:**
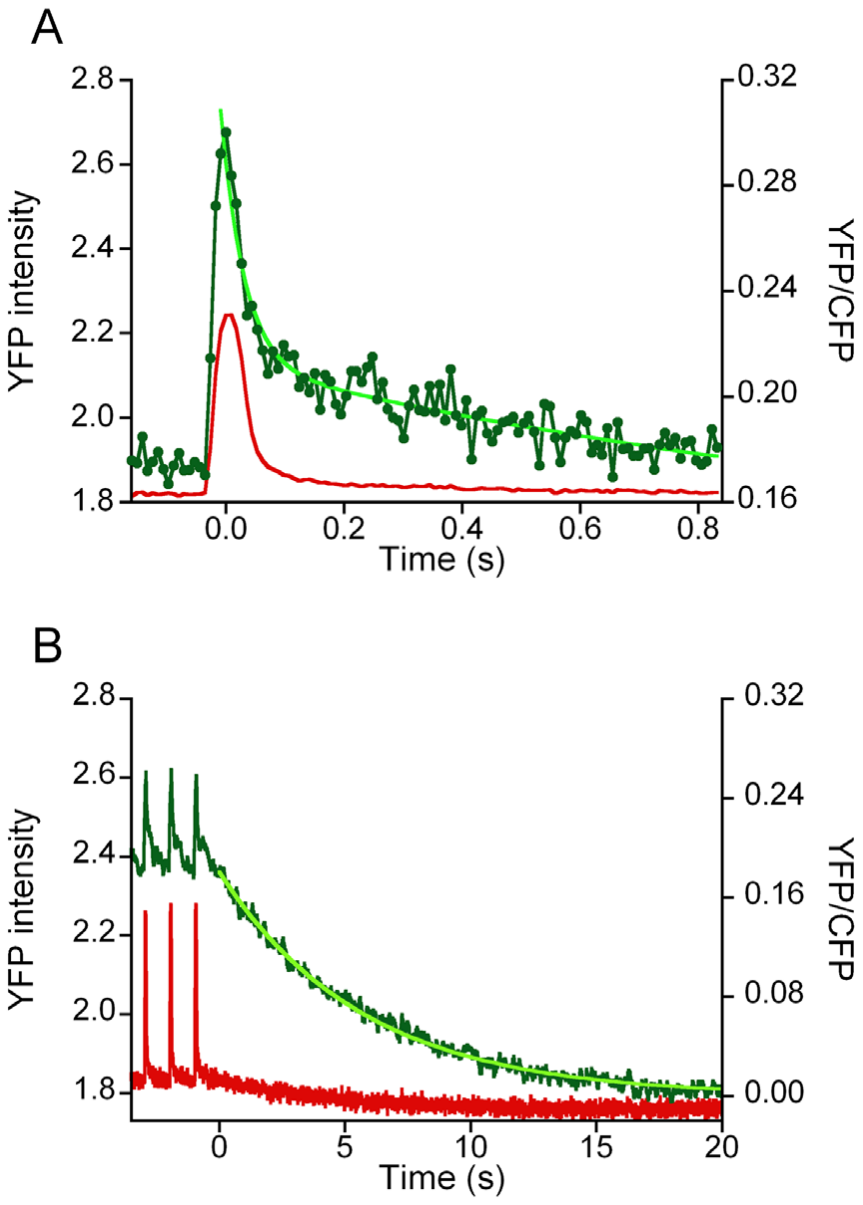
Time course of the decline in [Ca^2+^]_mito_. **A.** Time course of the variations in YFP/CFP ratio (green) *during* a train of stimuli at 1 Hz in a WT fiber. The time averaged response of the final 16 responses of the 20 s train of stimuli after subtraction of the baseline is shown. The time averaged YFP signal (red) is shown a surrogate marker of mechanical activity. A double exponential was fitted to the YFP/CFP data points R(t) = a_0_+a_1_. exp (−k_1_. t) + a_2_exp (−k_2_. t). The parameter values of the recording shown are: a_0_ = 0.149, a_1_ = 0.107, k_1_ = 39.8 s^−1^; a_2_ = 0.068, k_2_ = 1.15 s^−1^. **B.** Recording of the final decay of the (baseline subtracted) YFP/CFP ratio *after* a stimulus train at a frequency of 1 Hz. The time course of the decrease in YFP/CFP ratio was dominated by a single exponential (in bright green): R(t) = a_3_. exp (−k_3_.t), with amplitude (a_3_) and rate constant (k_3_) of 0.175 and 0.21 s^−1^, respectively.

To obtain more information on the kinetics of the intial phase of the decay in mitochondrial [Ca^2+^], we studied the averaged steady state recordings obtained during 1 Hz stimulation. At this stimulation rate, cytosolic calcium transients and contractile responses are well separated by periods at resting calcium concentration, while the free [Ca^2+^]_mito_ remained well above the resting initial values (see for example **Fig S4A in File S1**). Averaging of the steady state responses as well as pooling the results obtained in different fibers from the same experimental group allowed us resolve changes in the sub-millisecond range. A typical example is shown in **Fig. 6A**. The decline in the YFP/CFP ratio and hence [Ca^2+^]_mito_ during the 1 Hz stimulation period was fitted to a double exponential as indicated in Methods. The decay in free [Ca^2+^]_mito_ appeared to be dominated by two exponentials with a very fast rate constant (k_1_) of about 40 s^−1^ and a slower rate (k_2_) of about 1.5–2 s^−1^.

Recovery of the free [Ca^2+^]_mito_ after the end of a train of stimuli was dominated by a single exponential with a rate constant (k_3_) of approximately 0.20–0.25 s^−1^ (see **Fig. 6B**). The recordings at 20 and 60 Hz revealed an initially fast recovery with relatively small amplitude which resembled the fast decay during the 1 Hz stimulation period. However, the main component of the decline after the train of stimuli did not depend on stimulation frequency as can be appreciated also comparing the panels of **Fig. 3**. Accordingly, the results obtained at different stimulation frequencies were pooled. Small (∼10%) but significant (P<0.05) differences were observed in k_3_ between WT and dCASQ-null, indicating that recovery of free [Ca^2+^]_mito_ at the end of a train of stimulation in dCASQ-null fibers was faster than in WT fibers (see **Table** **1**).

**Table 1:** Parameter values of the exponential equations describing the recovery of [Ca^2+^]_mito_ during and after 1 Hz stimulation.

Group	Very fast phase		F Fast phase			Slow phase		
	a_1_	k_1_	a_2_	k_2_	n	a_3_	k_3_	n
**WT + Ca**	0.150±0.011	38.8±2.6	0.119±0.006	1.48±0.06	50	0.250±0.015	0.226±0.008	58
**WT-Ca**	0.125±0.016	32.8±3.1	0.125±0.019	1.26±0.11	18	0.190±0.023*	0.225±0.007	23
**dCASQ-null + Ca**	0.093±0.009	39.2±3.4	0.103±0.006	1.92±0.10	22	0.158±0.013	0.269±0.011	27
**dCASQ-null-Ca**	0.105±0.067	25.1±10.9	0.087±0.045	1.15±0.25*	2	0.122±0.028	0.235±0.026	4
**ANOVA**	ns	ns	ns	Ca^2+^: P<0.01	-	Ca^2+^: P<0.01 Type: P<0.001	ns^¶^	-

Amplitude (a_1_, a_2_ and a_3_) and rate constants (k_1_, k_2_ and k_3_ in s^−1^) of the recovery of [Ca^2+^]_mito_ during and after 1 Hz stimulation (n =  number of fibers). ^*^P<0.05: (−Ca)-value vs. corresponding (+ Ca)-value (Bonferroni post-hoc test). ^¶^Averaged rate constants of slow phase derived from all stimulation frequencies were type-dependent (P<0.05, see text).

**Figure 7:**
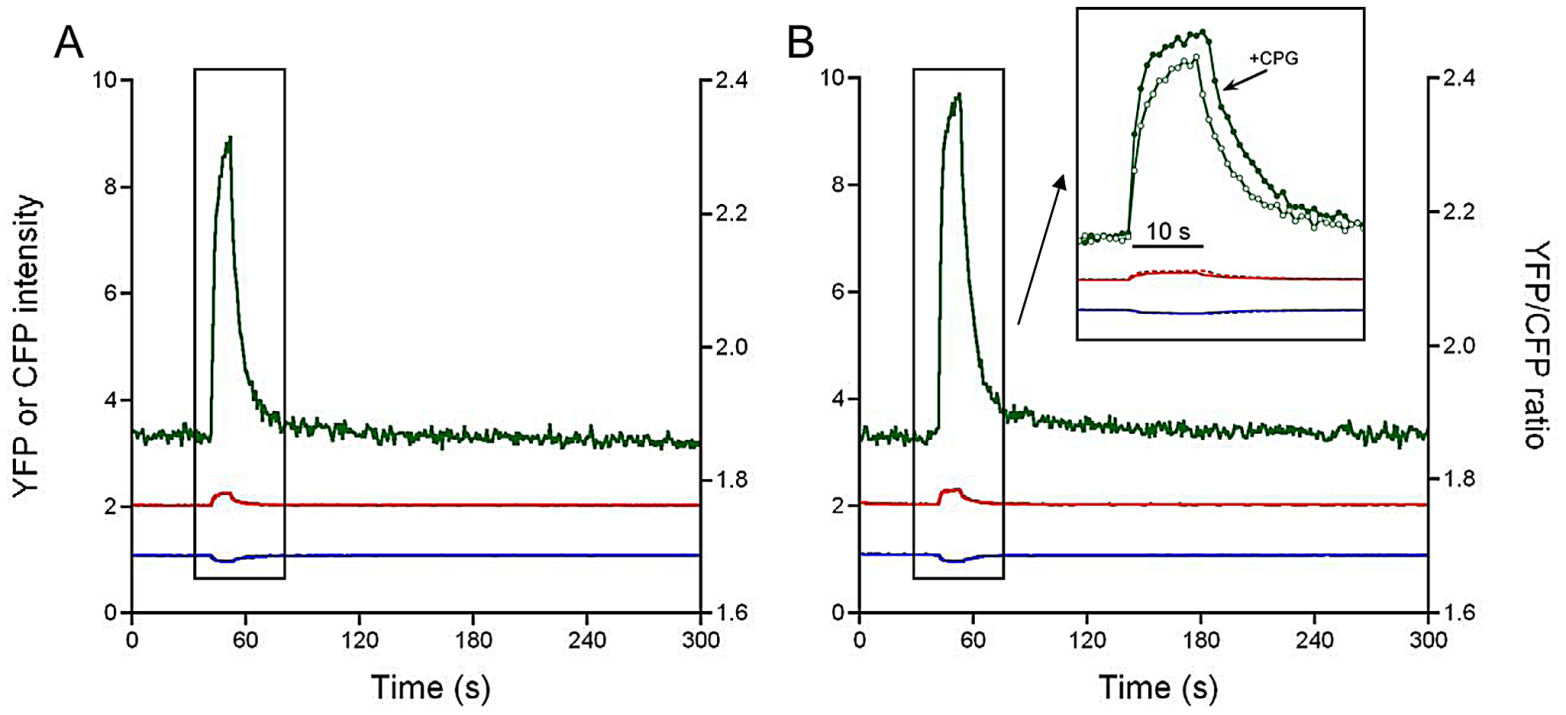
Effect mNCX blockade on the increase in [Ca^2+^]_mito_ during repetitive stimulation at 5 Hz. **A.** The 4mtD3cpv response (YFP, red; CFP, blue; ratio YFP/CFP, green) recorded at low illumination intensity to avoid bleaching of the probe in a WT fiber in the presence of 1 mM extracellular Ca^2+^. **B.** Response obtained in the fiber in the presence of 1 μM CGP37157 (CGP: a selective mitochondrial Na^+^/Ca^2+^ exchanger blocker). In the inset a comparison is shown of the recordings +/− CGP37157 on an expanded time scale, illustrating that in the presence of CGP37157 [Ca^2+^]_mito_ rises faster and reaches a higher final level. This is consistent with blockage of mitochondrial efflux via the mNCX during the stimulation period (10 s).

To quantitate possible very slow components of the [Ca^2+^] decay process we extended the recording of the decay phase after a train of 5 Hz stimulation to 10 minutes. In these recordings the illumination intensity was reduced accordingly in order to reduce bleaching of the probe. The time resolution of the recording amounted to 900 ms. The results obtained in 2 WT and 2 dCASQ-null fibers indicated the presence of an additional, very slow component. The relative amplitude (a_4_) and the rate constant (k_4_) of this component amounted to 12±4% and 0.060±0.005 s^−1^ and 9±2% and 0.014±0.010 s^−1^, in WT + Ca and dCASQ-null + Ca fibers, respectively. In these experiments, the effects of CGP37157 a specific inhibitor of the mitochondrial Na^+^/Ca^2+^ exchanger were also tested. It can be seen in **Fig. 7** that addition of 1 μM CGP37157 resulted in a more abrupt rise in [Ca^2+^]_mito_ and an increase in the final level reached, during the 10 s period of 5 Hz stimulation.

An overview of the data obtained with 1 Hz stimulation in WT and dCASQ-null fibers in the presence or absence of extracellular Ca^2+^ is shown in **Table** **1**. In general, the parameter values observed in dCASQ-null -Ca group showed more variability than the responses in the other groups, most likely because of larger variability in Ca^2+^ content of intracellular stores in dCASQ-null fibers in the absence of extracellular Ca^2+^. It can be seen that the amplitude of the slow (k_3_≈0.25 s^−1^) component (a_3_) in WT + Ca fibers was larger than the amplitude of the fast (a_2_) and the very fast component (a_1_). Highly significant differences were observed in a_3_ as well as in the rate constant of the fast component (k_2_). The differences observed in a_3_ were both type- (WT vs. dCASQ-null; P<0.001) as well as Ca^2+^-dependent (1 mM vs. 0 mM external Ca^2+^; P<0.01). This latter observation is in line with the data reported in Fig.****4 and suggests the mitochondrial Ca^2+^ concentration was reduced even in WT fibers in the absence of external Ca^2+^. The differences in k_2_ were Ca^2+^-dependent (P<0.01) but not type-dependent. In WT-Ca fibers, the amplitude of a_3_ was significantly smaller than in WT + Ca fibers (P<0.05). Post-hoc analysis revealed that the rate constant k_2_ in the absence of extracellular Ca^2+^ was significantly reduced (P<0.05). The type-dependent difference in the rate constant k_3_ which is significant when the values obtained at all stimulation rates are pooled together (see above), is visible as a trend but did not reach statistical significance in the results using 1 Hz stimulation only.

In summary, our results imply that overall mitochondrial Ca^2+^ decay can be described by (at least) 4 different exponentials (very fast, fast, slow and very slow) with clearly distinct rate constants (k_1_, k_2_, k_3_ and k_4_) of approximately 40, 1.5, 0.25 and 0.03 s^−1^. The amplitudes and rates of several of these components turned out to differ between dCASQ-null and WT and to depend on the presence or absence of external Ca^2+^.

### Estimation of the free [Ca^2+^]_mito_

The actual values of the free [Ca^2+^]_mito_ were derived from the equation: [Ca^2+^]  = β·K'_d_·[(R-R_min_)/(R_max_-R)]^1/n^ [22], using average R_max_ and R_min_ values of 5.83 and 1.50, respectively, an *in vitro* K'_d_ of the 4mtD3cpv sensor of 0.76 μM, an n value of 0.74 [22] and a value for β of 6 [30]. The values obtained in the presence of extracellular Ca^2+^ in quiescent fibers amounted to 0.16 μM (WT) and 0.23 μM (dCASQ-null) and to a maximum free [Ca^2+^]_mito_ at the end of the train of stimuli at 20 or 60 Hz of 1.10 μM (WT) and 0.73 μM (dCASQ-null).

### Cytosolic Ca^2+^ transients during and after repetitive stimulation

To assess the relation between free cytosolic [Ca^2+^] (or [Ca^2+^]_cyto_) and the free [Ca^2+^]_mito_, we determined free [Ca^2+^]_cyto_ with the fluorescent indicator Fura-2. In quiescent fibers, no significant differences were observed in the free [Ca^2+^]_cyto_ between WT and dCASQ-null fibers both in the absence and presence of extracellular Ca^2+^. The free [Ca^2+^]_cyto_ of the pooled data amounted to 101±2 nM (n = 90).

**Figure 8:**
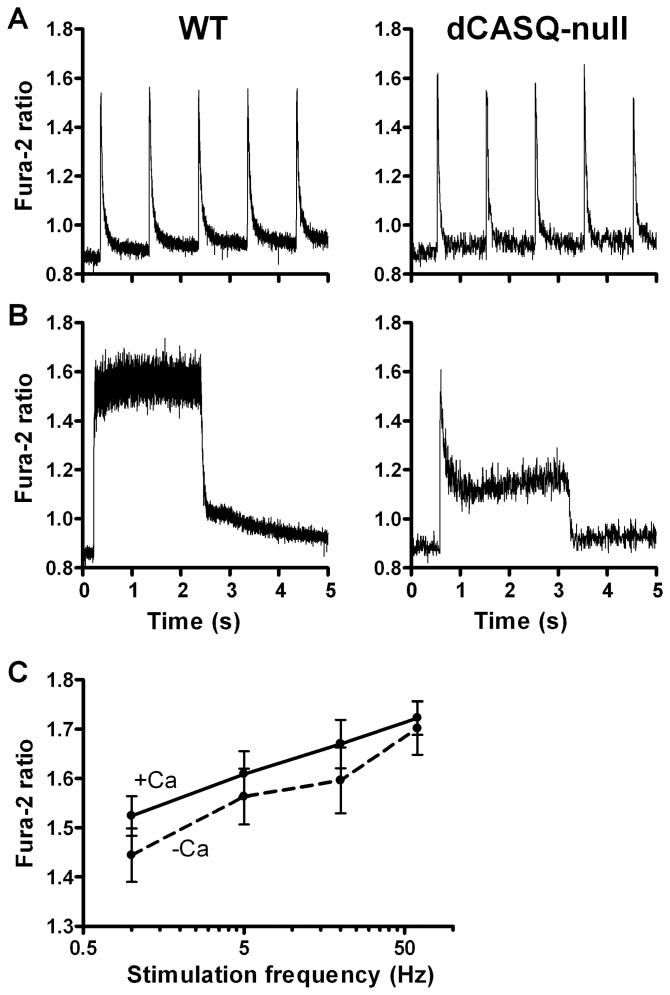
Cytosolic Ca^2+^ concentration in electrically stimulated WT and dCASQ-null fibers. **A.** At low frequency of stimulation (1 Hz) Fura-2 transients have comparable amplitudes in WT and dCASQ-null fibers and, due to their short duration, are intercalated by periods of low resting cytosolic Ca^2+^ levels. **B.** At high frequency of stimulation (60 Hz) transients are fused and Ca^2+^ concentration remains high in WT, while it declines after an initial peak in dCASQ-null fibers. **C.** Average values of peak cytosolic Ca^2+^ concentration probed by Fura-2 ratio at the end of stimulation trains at various stimulation frequency in WT, in the presence (triangles) or absence (circles) of extracellular Ca^2+^.

The recovery of the cytosolic Ca^2+^ transient was virtually complete at 1 Hz stimulation frequency (**Fig. 8A** **and Fig. S4 in File S1**), but at 5 and 60 Hz (**Fig. 8B** **and Fig. S4 in File S1**), an increase in the baseline was observed during stimulation. In WT fibers immersed in 1 mM Ca^2^, in agreement with the results of previous studies, e.g. [31], the peak values of the Ca^2+^ transients during repetitive stimulations increased with stimulation frequency (**Fig. 8C**). In the absence of extracellular Ca^2+^ the increase in peak values during the repetitive stimulations was very similar (P = 0.47). In dCASQ-null fibers, even in the presence of external Ca^2+^, a marked decline followed the initial peak in Ca^2+^ concentration reached during stimulation (**Fig. 8B** **and Fig. S4 in File S1)**. In the absence of extracellular Ca^2+^, the amplitude of the Ca^2+^ transients decayed rapidly with subsequent stimulation at 1 Hz and hardly any increase in [Ca^2+^]_cyto_ was observed during repetitive stimulations at 5, 20 and 60 Hz, pointing to a rapid depletion of the SR [17]. In those fibers the amplitude of the Fura-2 transient was quite variable and on average much smaller than in the presence of extracellular Ca^2+^.

## Discussion

The use of a highly specific ratiometric probe indicates that electrical stimulation of a muscle fiber is followed by a mitochondrial Ca^2+^ transient with a rise time of 20–30 ms and a decay phase consisting of (at least) 4 different components, with distinct rate constants. The comparison between WT fibers and dCASQ-null fibers revealed significant differences in mitochondrial calcium concentration at rest and during contraction and in the kinetics of both the rising and the falling phases of the mitochondrial Ca^2+^ transient. These results suggest that the mitochondrial calcium handling is affected by the amount of calcium stored in the SR. In addition, our results indicate that mitochondrial Ca^2+^ handling is sensitive to Ca^2+^ entering into the fibers from the extracellular space. Our results also confirm and extend previous observations on alterations in mitochondrial volume and in the coupling of the CRU's to the mitochondria in dCASQ-null mice, a model of malignant hyperthermia, and suggest that these alterations may be adaptive.

### Free mitochondrial [Ca^2+^] in quiescent muscle fibers

In quiescent fibers free [Ca^2+^]_mito_ in dCASQ-null fibers is significantly higher than in WT fibers, as indicated by the difference in the basal YFP/CFP ratios. Our estimates of the free [Ca^2+^]_mito_ are 0.16 μM in WT and 0.23 μM in dCASQ-null fibers. These values are very similar to previous estimates of 0.2 μM in WT, see e.g. [32]. It should be noted, however, that our estimates of the free [Ca^2+^]_mito_ are rather crude because of uncertainties in the (in situ) values of K'_d_ and β of the probe used in our experiments.

Evidence obtained in skeletal and cardiac muscle cells suggests a free/bound Ca^2+^ proportion inside mitochondria of 1/100 [32], [33], thus implying a total concentration of Ca^2+^ stored inside the mitochondria at rest of 16 μM in WT fibers and 20 μM in dCASQ-null fibers. The intermyofibrillar mitochondrial volume fraction was increased from 4.3±0.1% in WT to 7.2±0.2% in dCASQ-null fibers, i.e. by 67±6%. The intermyofibrillar mitochondria represent a major fraction of the whole population which includes subsarcolemmal and perinuclear mitochondria [34]. Thus, our estimate of the overall mitochondrial volume fraction in WT fibers is less than half of the value of 9.9% obtained from fluorescence measurements in murine FDB fibers [35]. The reason for this difference is unclear but it can be noted that different techniques were used to visualize the mitochondria. Assuming an overall mitochondrial volume fraction of 4.3% in WT and 7.2% in dCASQ-null fibers, the mitochondrial Ca^2+^ content expressed per fiber volume would correspond to 0.7 μmol/L in WT fibers and to 1.7 μmol/L in dCASQ-null fibers. This increase in mitochondrial Ca^2+^ concentration at rest in dCASQ-null relative to WT could be related to the increase of Ca^2+^ leakage from the SR lacking calsequestrin [36].

### Free mitochondrial [Ca^2+^] in contracting muscle fibers

Our results indicate that the mitochondrial Ca^2+^ transient is highly asymmetric with a short rising phase (rise time: 20–30 ms) and a much longer decay phase which can be described by (at least) 4 different exponentials, with clearly distinct rate constants of 40, 1.5, 0.25 and 0.03 s^−1^. These results add quantitative evidence to earlier findings that the free mitochondrial Ca^2+^ concentration increases when SR Ca^2+^ release is triggered by action potentials [8] and leads to accumulation of Ca^2+^ inside mitochondria during sustained muscle activity [8], [35].

Our estimations yield to maximal levels of free Ca^2+^ concentration reached during repetitive stimulation of 1.10 and 0.73 μM, in WT and dCASQ-null fibers, respectively. Taking the difference in mitochondrial volume fraction into account (see above), the total amount of Ca^2+^ inside the mitochondrial expressed per cell volume during contraction would correspond to 4.71 and 5.27 μmol/L, in WT and in dCASQ-null fibers, respectively. Assuming that the total calcium release ranges from 200–300 μmol/L after a single action potential to 400–500 μmol/L after repeated stimulation [37] mitochondrial Ca^2+^-uptake corresponds to approximately 1% of the total amount of Ca^2+^ released into the cytosol. A similar value has been calculated in cardiomyocytes [33], while determinations in skeletal muscle fibers range from very low values [35], [38] to higher values as the recent estimate of 10–18%, derived under voltage clamp conditions in skeletal muscle fibers [7]. This implies that the contribution of mitochondrial Ca^2+^-uptake in fast muscle fibers would be rather small but not negligible, as was concluded previously [39]. However, the small fraction taken up by the mitochondrial could still be highly relevant for the regulation of key metabolic enzymes and the F0-F1 ATPase.

### Kinetics of the rising phase of the mitochondrial Ca^2+^ transient

No differences were observed in the kinetics of the rising phase of the mitochondrial Ca^2+^ transient between WT and dCASQ-null. The average rise time of the increase in [Ca^2+^]_mito_ (t_10–90%_) amounted to 27.8±1.6 ms during the first contraction in a rested fiber and to 18.5±0.4 ms during steady state contractions at 1 Hz. Since the rise time is influenced by the intrinsic response time of the probe, the actual rise time of mitochondrial Ca^2+^-uptake might even be faster. The agreement between values obtained in WT and dCASQ-null fibers indicates that it is not significantly influenced by a reduction in the SR storage capacity, induced by ablation of both CASQ isoforms, possibly because the local cytosolic calcium concentrations during the initial contrations in WT and dCASQ-null are similar.

Since the cytosolic Ca^2+^ transients during the initial contraction and the steady state contractions at 1 Hz are very similar (see **Fig. S4 in File S1**), the difference in the rise time in rested fibers and during steady state at 1 Hz is in fact opposite to what would be expected in a situation where substantial Ca^2+^-accumulation in the mitochondria would have taken place. However, the speed of mitochondrial Ca^2+^-uptake process could be increased as a result of a rise the local Ca^2+^-concentration in the microdomains between CRUs and mitochondria during repetitive stimulation at 1 Hz. In order to be consistent with Ca^2+^-uptake through the low affinity MCU, this local Ca^2+^-concentration would need to be increased considerably above the cytosolic free Ca^2+^-concentration at rest (∼100 nM). Evidence suggests that the Ca^2+^ uniporter can be sensitized by the locally elevated [Ca^2+^], see e.g. [3] and estimates of local [Ca^2+^] in cardiac muscle cells, up to 10 μM [40], are also consistent with this possibility.

A small but significant prolongation of the rising phase of mitochondrial Ca^2+^ transient during 1 Hz stimulation was observed in dCASQ-null in the absence of external Ca^2+^. Under these conditions the intracellular stores are almost exhausted and unable to support a prolonged contractile activity [17]. It is likely therefore that the prolongation is caused by limited Ca^2+^ availability in the cytosol rather than a direct specific effect of extracellular Ca^2+^ on mitochondrial Ca^2+^-uptake kinetics per se.

### Kinetics of the decay phase of the mitochondrial Ca^2+^ transient

The decline in [Ca^2+^]_mito_ during and after stimulation trains is governed by 4 exponentials with rate constants of approximately 40, 1.5, 0.2 and 0.03 s^−1^ (at 26°C). The amplitude of the very fast component amounted to 25–30% of total Ca^2+^ release both in WT as in dCASQ-null fibers (**Table** **1**). The occurrence of rapid uptake and release processes simultaneously may explain why previous attempts to determine mitochondrial Ca^2+^ uptake, with lower time resolution, yielded contrasting results [41].

Selective blockade of the mNCX by CGP37157 (**Fig. 6**) enhanced the rise in free [Ca^2+^]_mito_ and increased the final level attained at the end of 10 s stimulation train. CGP37157 had no effect on the rise time of the increase in [Ca^2+^]_mito_ nor on the fast rate of the Ca^2+^-recovery process (results not shown). Assuming that the free [Ca^2+^]_mito_ is the result of a balance between Ca^2+^ influx and efflux across mitochondrial inner membrane [42], these findings suggest that the mitochondrial Na^+^/Ca^2+^ exchanger promotes Ca^2+^ efflux already during the stimulation period. Since the effect was visible on a 10 s time scale we consider it likely that the mNCX may contribute to the intermediate (1.5 and 0.3 s^−1^) phases of the mitochondrial Ca^2+^-extrusion process.

### Differences in mitochondrial structure and Ca^2+^ handling between WT and dCASQ-null fibers

Analysis of the EM-images indicated that in dCASQ-null fibers the mitochondrial volume was increased by 67%, the percentage of mitochondria coupled to the CRUs was increased by 12% and that the width of the terminal cisternae of the SR was reduced in comparison to WT. These findings and the increased [Ca^2+^]_mito_ at rest imply that the total amount of Ca^2+^ stored in mitochondria is increased, as discussed above, and that the exchange of Ca^2+^ between the SR and mitochondria could be facilitated by an improved coupling and a shortened Ca^2+^ diffusion distance between the sites of release and the mitochondria [10]. Assuming that the increased Ca^2+^ leakage from the SR results in an elevation of the Ca^2+^ concentration near the mitochondria relative to the concentration in the bulk of the cytosol, the improved coupling with SR and the increased Ca^2+^ leakage from SR could explain the increase in free [Ca^2+^]_mito_ in quiescent dCASQ-null fibers relative to WT fibers, whereas global free cytosolic Ca^2+^ concentration remained the same. Moreover, it can be noted that the intermyofibrillar mitochondria are located close to the regions of the SR where SERCA is localized (see **Fig. 2**). Thus, leakage through SERCA in the absence of calsequestrin could play a specific role as previously suggested [36]. The increased calcium concentration in the mitochondrial matrix might play a role in increasing the metabolic activity. The greater activity of the mitochondrial metabolic machinery at rest might, in turn, lead to greater production of ATP and reactive oxygen species (ROS).

The steady levels of the free mitochondrial Ca^2+^ concentration attained during the repetitive contractions were lower in dCASQ-null than in WT. This reduction can be attributed to the depletion of SR and to the progressive decay of cytosolic Ca^2+^ concentration during the contractions as a result of Ca^2+^-binding to parvalbumin (see **Fig. 7** **and Fig. S4 in File S1**) and [17], [19]. Interestingly, this reduction in Ca^2+^-accumulation is predominantly reflected during the decline phase in a reduction of the amplitude of the slow recovery process (a_3_). Thus, the amplitude of this latter phase appears to be particularly sensitive to the reduced mitochondrial Ca^2+^-content of dCASQ-null fibers in comparison with WT fibers. This observation is compatible with the idea that the speed of this phase is determined at least partly by the Ca^2+^-off rate(s) of mitochondrial Ca^2+^ buffer(s).

### Mitochondrial Ca^2+^-handling in the absence of extracellular Ca^2+^

Our previous study [17] showed a rapid loss of contractile response of dCASQ-null fibers after incubating them in a solution without Ca^2+^, indicating that sarcolemmal Ca^2+^-influx possibly via SOCE occurred during stimulation and was required to support the activation of the contractile process in those fibers. Our present results indicated that the elevated free mitochondrial [Ca^2+^] in quiescent dCASQ-null fibers was maintained in the absence of external Ca^2+^. We attribute this to enhanced coupling between the SR and the mitochondria because the cytosolic Ca^2+^ concentration at rest: 1) did not differ between WT and dCASQ-null fibers and 2) was not significantly affected by external Ca^2+^ removal.

Interestingly, the rate constant (k_2_) of the fast Ca^2+^ extrusion phase is reduced in dCASQ-null fibers when extracellular Ca^2+^ is removed, back to WT values. This suggests that in addition to improved coupling between CRUs and mitochondria in dCASQ-null fibers, the activity of the mNCX could be reduced in these fibers in the absence of extracellular Ca^2+^, e.g. by a rise in mitochondrial Na^+^ concentration [43].

### Conclusions and implications

This study represents the first detailed quantitative analysis of the kinetics of mitochondrial Ca^2+^ handling in fast skeletal muscle fibers. It extends previous data on mitochondrial Ca^2+^ handling in skeletal muscle fibers [8], [10], [35], [44]. In addition, our results provide evidence of the dependence of [Ca^2+^]_mito_ on intracellular and external Ca^2+^ stores and reveal the presence of multiple processes of free mitochondrial Ca^2+^ buffering or extrusion operating at a time scale ranging from milliseconds to minutes. Our analysis also provides direct evidence for the involvement of the mitochondrial Na^+^/Ca^2+^ exchanger (mNCX) in mitochondrial Ca^2+^ extrusion process, relevant at the intermediate time scale (seconds or tens of seconds).

Rapid Ca^2+^ uptake and release by the mitochondria play an important but still under-illuminated role in the regulation mitochondrial metabolic activity. Our study indicates that mitochondrial Ca^2+^-uptake and release are fast enough to allow the rapid adjustment of metabolic activity to increased demand for instance during exercise. Moreover, Ca^2+^ uptake by the mitochondria is crucial in skeletal and cardiac dysfunction and in programmed cell death, e.g. [3], [4].

Calsequestrin-null mice represent a model for malignant hyperthermia [21], [45]. The number of mitochondria and the resting free Ca^2+^ concentration inside the mitochondria were increased in calsequestrin-null muscle and the maximum free Ca^2+^ levels reached during electrical stimulation were sensitive to the presence of extracellular Ca^2+^. These alterations in mitochondrial Ca^2+^ handling may represent a compensatory response when SR Ca^2+^-content is reduced, e.g. by calsequestrin ablation and/or increased SR Ca^2+^ leakage, but also may render the tissue more prone to the development of contractures.

## Supporting Information

File S1 **Supporting text and figures. Figure S1. In situ determination of R_min_ (left) and R_max_ (right) of the cameleon (4mtD3cpv).** The 4mtD3cpv responses (YFP, red; CFP, blue; ratio YFP/CFP, green) are shown. Aliquots of ionomycin (Iono) were added up to a final concentration of 5 μM (left) and 10 μM (right); 50 μM N-benzyl-ptoluene sulphonamide (BTS) was added in order to reduce movement artifacts during the R_max_ determination. The minimum YFP/CFP value reached (R_min_) amounted to 1.5. Addition of 5 mM CaCl_2_ caused an abrupt increase in the YFP/CFP ratio up to a maximum value of 6.3. Upon the addition of 5 mM CaCl_2_, the fiber started to contract despite of the presence of BTS (for 7 minutes), resulting in an increase in the CFP (and YFP) intensity and moved out of the focal plane of the microscope near the end of the recording (resulting in a decline in the YFP and CFP intensity). **Figure S2. Correction for bleaching.** Top: during a train of stimuli at 0.1 Hz, the amplitude of the increase in the YFP/CFP ratio declined as a result of bleaching of the probe. Bottom: a linear relation was observed between peak amplitude and the concurrent baseline value, yielding a straightforward way to correct the ΔR values for bleaching of the probe (see Results). **Figure S3. Recording of the change in [Ca^2+^]_mito_ during and after a single twitch.** The baseline corrected 4mtD3cpv response (YFP, red; CFP, blue; ratio YFP/CFP, green) in a WT fiber electrically stimulated by a single pulse in the presence of 1 mM Ca^2+^. The YFP/CFP ratio shown was obtained by using a 10-points running average. The final part of the decay phase could be well fitted to a single exponential (bright green) with a rate constant of 0.21 s^−1^. **Figure S4.** **Comparison of the time course of the 4mtD3cpv responses and the Fura-2 responses.** In each panel, the upper figure shows the 4mtD3cpv response and the lower figure shows the Fura-2 response in WT at 1, 5 and 60 Hz stimulation (A, B and C) and in dCASQ-null at 60 Hz (D). Note that the decline in the 4mtD3cpv transients at 1 and 5 Hz occurs more slowly than in the Fura-2 transients. As a result there is -during the stimulation period- a gradual rise in the baseline free Ca^2+^ concentration inside the mitochondria, whereas the cytosolic Ca^2+^ concentration in between stimuli remains rather constant (Panels A and B). The comparison between WT and dCASQ-null at 60 Hz illustrates that in dCASQ-null fibers the initial peak value is similar to WT but there is a gradual decline in the cytosolic free Ca^2+^ concentration. At 60 Hz stimulation, the free [Ca^2+^]_mito_ after an early rapid increase accumulates gradually in WT but remains rather constant in dCASQ-null fibers (Panels C and D).
(DOC)
