# Supplementary material for: Mitochondrial Ca2+-Handling in Fast Skeletal Muscle Fibers from Wild Type and Calsequestrin-Null Mice
Source: PLoS One. 2013 Oct 3;8(10):e74919. doi: 10.1371/journal.pone.0074919 (PMC3789688; doi:10.1371/journal.pone.0074919)
Supplement: File S1 — Supporting text and figures. Figure S1. In situ determination of Rmin (left) and Rmax (right) of the cameleon (4mtD3cpv). The 4mtD3cpv responses (YFP, red; CFP, blue; ratio YFP/CFP, green) are shown. Aliquots of ionomycin (Iono) were added up to a final concentration of 5 μM (left) and 10 μM (right); 50 μM N-benzyl-ptoluene sulphonamide (BTS) was added in order to reduce movement artifacts during the Rmax determination. The minimum YFP/CFP value reached (Rmin) amounted to 1.5. Addition of 5 mM CaCl2 caused an abrupt increase in the YFP/CFP ratio up to a maximum value of 6.3. Upon the addition of 5 mM CaCl2, the fiber started to contract despite of the presence of BTS (for 7 minutes), resulting in an increase in the CFP (and YFP) intensity and moved out of the focal plane of the microscope near the end of the recording (resulting in a decline in the YFP and CFP intensity). Figure S2. Correction for bleaching. Top: during a train of stimuli at 0.1 Hz, the amplitude of the increase in the YFP/CFP ratio declined as a result of bleaching of the probe. Bottom: a linear relation was observed between peak amplitude and the concurrent baseline value, yielding a straightforward way to correct the ΔR values for bleaching of the probe (see Results). Figure S3. Recording of the change in [Ca2+]mito during and after a single twitch. The baseline corrected 4mtD3cpv response (YFP, red; CFP, blue; ratio YFP/CFP, green) in a WT fiber electrically stimulated by a single pulse in the presence of 1 mM Ca2+. The YFP/CFP ratio shown was obtained by using a 10-points running average. The final part of the decay phase could be well fitted to a single exponential (bright green) with a rate constant of 0.21 s−1. Figure S4. Comparison of the time course of the 4mtD3cpv responses and the Fura-2 responses. In each panel, the upper figure shows the 4mtD3cpv response and the lower figure shows the Fura-2 response in WT at 1, 5 and 60 Hz stimulation (A, B and C) and in dCASQ-null at 60 Hz (D). Note [file pone.0074919.s001.doc]

**SUPPORTING INFORMATION**

**Calibration of the cameleon (4mtD3cpv)**

In situ determination of R_min_ and R_max_ of the Ca^2+^ sensor was performed as describes previously (Palmer and Tsien; Nat Protoc 1:1057-1065, 2006) and is illustrated in **Fig. S1**. This protocol uses the Ca^2+^ ionophore ionomycin to render all membranes permeable to Ca^2+^ ions. In the absence of extracellular Ca^2+^ in the medium, this is expected to cause depletion of intracellular Ca^2+^ upon the addition of 3 mM EGTA allowing the determination of the minimum in the YFP/CFP ratio (R_min_). In the presence of 1 mM extracellular Ca^2+^, addition of ionomycin, the YFP/CFP ratio started to increase and upon addition of extra Ca^2+^ to the medium, the YFP/CFP ratio rapidly increased to a maximum value (R_max_).

The average R_min_ and R_max_ values determined in different fibers amounted to 1.50±0.03 (n=4) and 5.83±0.26 (n=6), respectively.

**
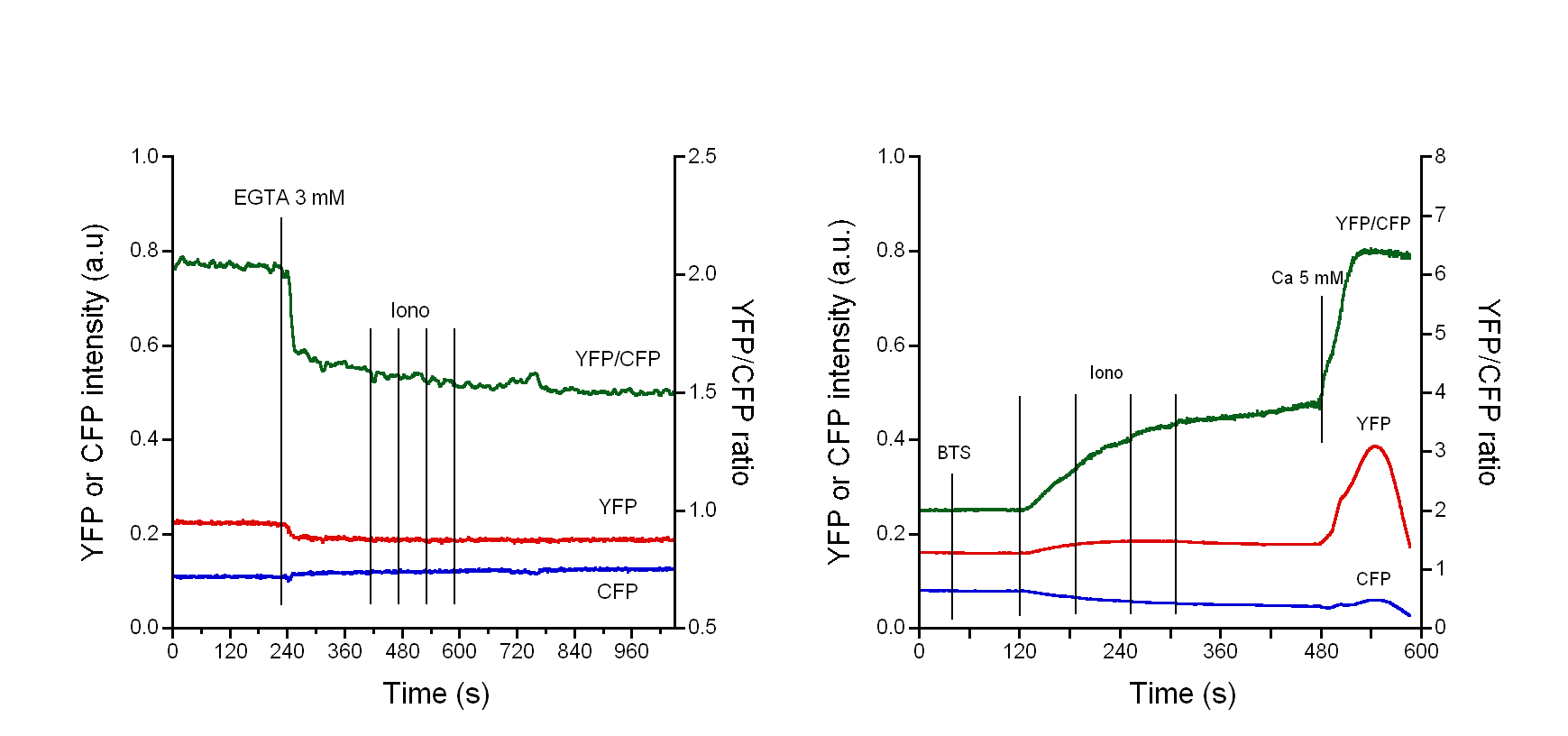
Figure S1. In situ determination of R_min_ (left) and R_max_ (right) of the cameleon (4mtD3cpv).** The 4mtD3cpv responses (YFP, red; CFP, blue; ratio YFP/CFP, green) are shown. Aliquots of ionomycin (Iono) were added up to a final concentration of 5 μM (left) and 10 μM (right); 50 μM N-benzyl-ptoluene sulphonamide (BTS) was added in order to reduce movement artifacts during the R_max_ determination. The minimum YFP/CFP value reached (R_min_) amounted to 1.5. Addition of 5 mM CaCl_2_ caused an abrupt increase in the YFP/CFP ratio up to a maximum value of 6.3. Upon the addition of 5 mM CaCl_2_, the fiber started to contract despite of the presence of BTS (for 7 minutes), resulting in an increase in the CFP (and YFP) intensity and moved out of the focal plane of the microscope near the end of the recording (resulting in a decline in the YFP and CFP intensity).

**Correction for bleaching**

The procedure to correct for bleaching of the probe is illustrated in the upper panel of **Fig. S2** in which a recording obtained in a WT fiber at 0.1 Hz stimulation for a period of about 3 minutes is shown. The baseline and amplitude of the responses change in proportion as can be seen in the lower panel of **Fig. S2** where, the amplitude of the increases in [Ca^2+^]_mito_ are shown as a function of the baseline value of the YFP/CFP ratio at the time of electrical stimulation. It is clear that the relation is linear (R=0.994) and this allows for a relatively simple way to correct the change in the amplitude of the YFP/CFP ratio (ΔA) in the recordings for bleaching: ΔA_cor_ = ΔA/(1-C(R_o_-R_bl_)), in which R_o_ is equal to the initial YFP/CFP ratio at t=0 s in the fiber and R_bl_ equals the actual value of the baseline where ΔA is measured. The value of C, determined in 3 fibers amounted to 1.04±0.06.

To check the bleaching correction, recordings in WT fibers (n=6) were obtained at different stimulation frequencies when the illumination intensity was reduced by more than 32 times. The integration time of the camera was adjusted accordingly. This reduced the time resolution of the measurements but bleaching under these circumstances became negligibly small. The amplitudes of the increase in YFP/CFP ratio at low illumination intensity were very similar to the corrected values at high illumination intensity.


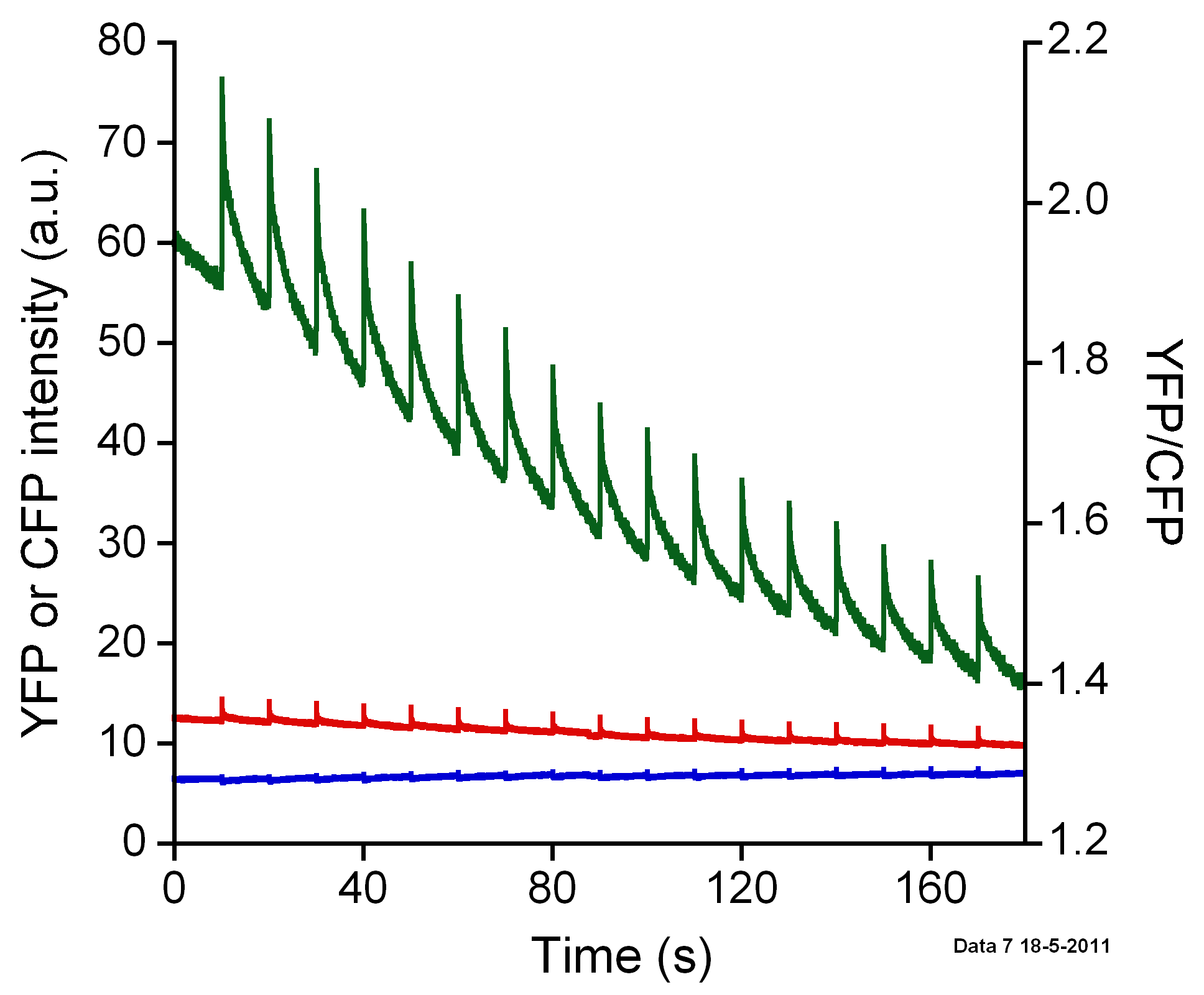

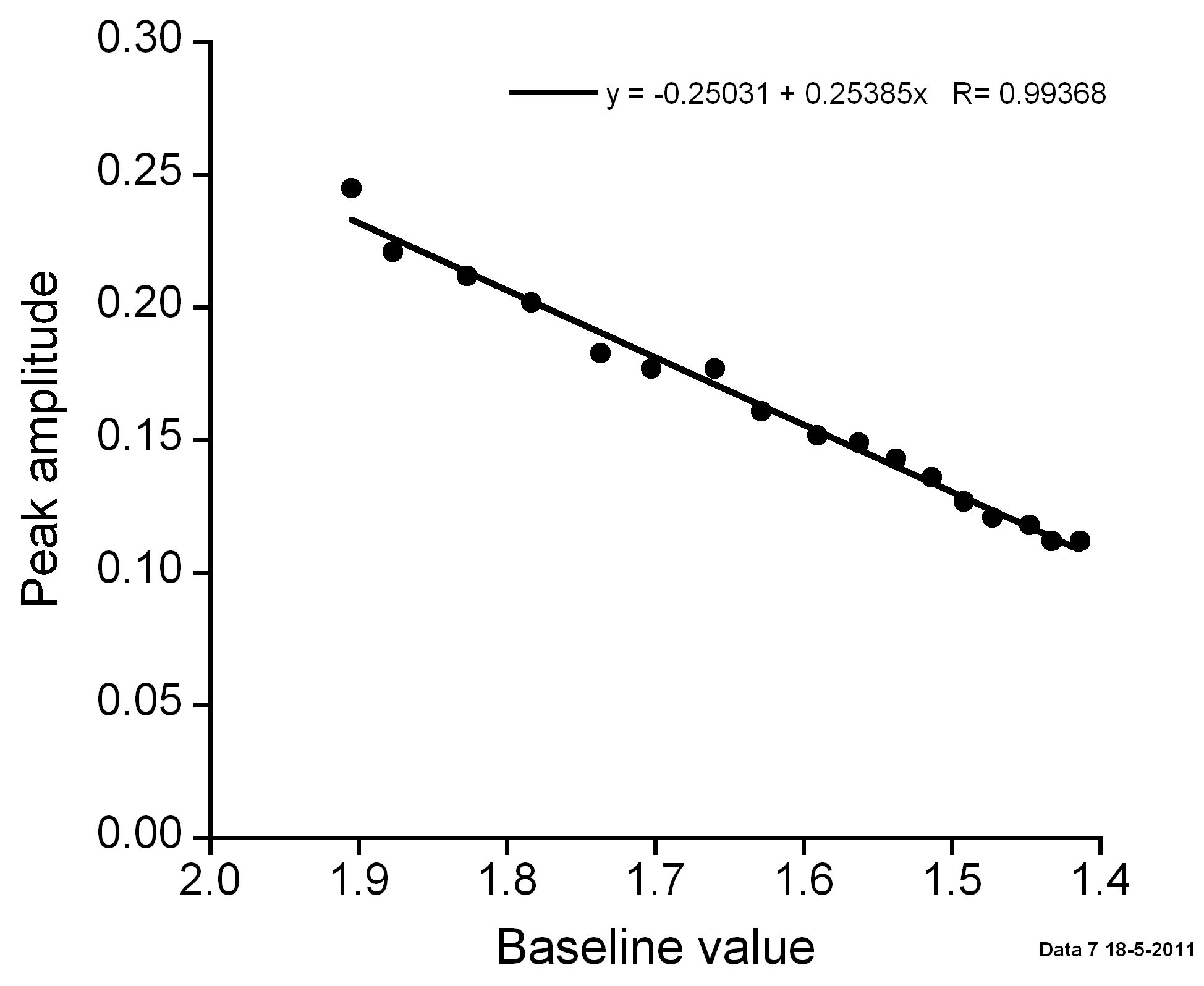


**Fig. S2. Correction for bleaching.** Top: during a train of stimuli at 0.1 Hz, the amplitude of the increase in the YFP/CFP ratio declined as a result of bleaching of the probe. Bottom: a linear relation was observed between peak amplitude and the concurrent baseline value, yielding a straightforward way to correct the ΔR values for bleaching of the probe (see Results).

**Change in [Ca^2+^]_mito_ during and after a single twitch**

**
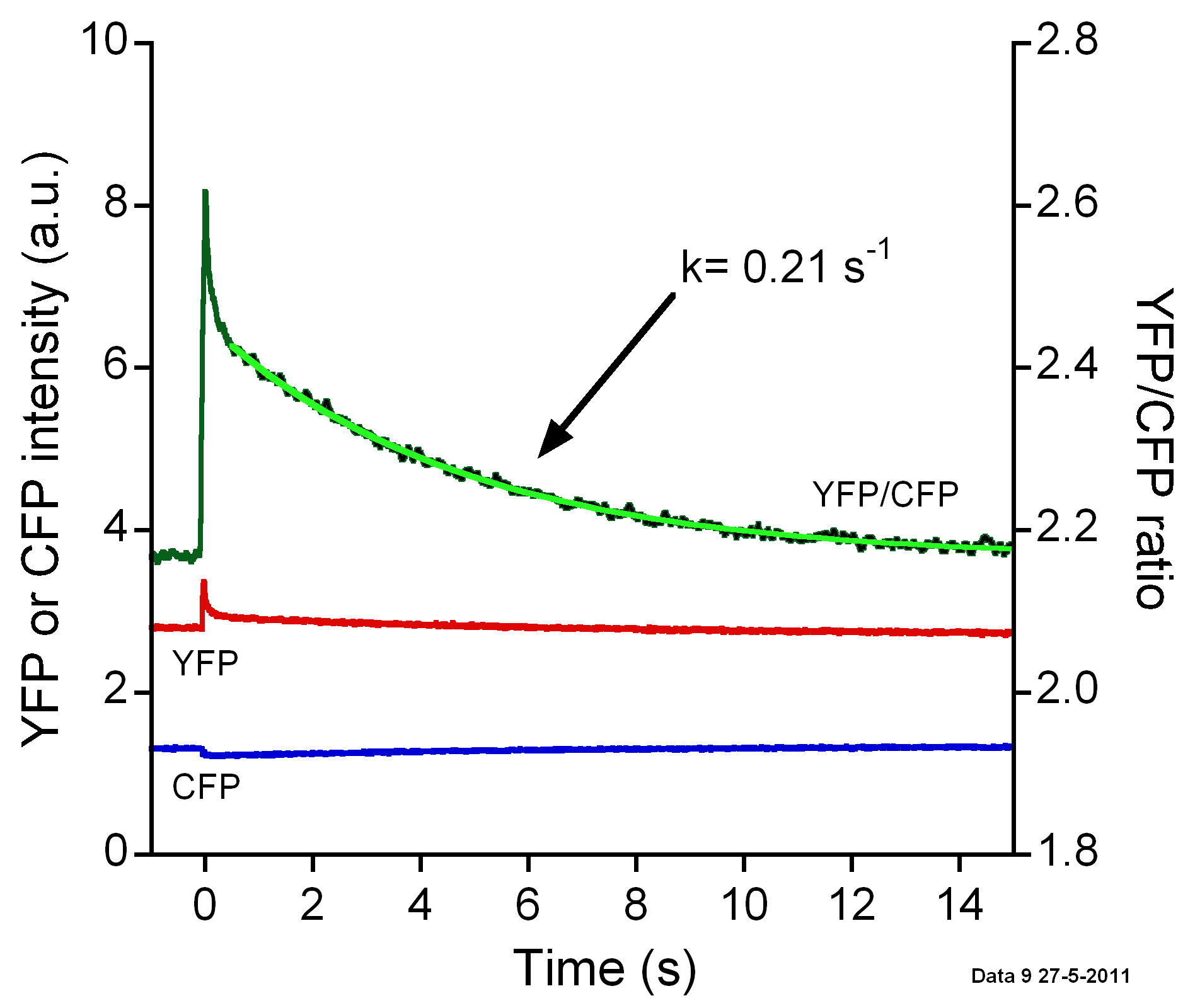
**

**Fig. S3. Recording of the change in [Ca^2+^]_mito_ during and after a single twitch.** The baseline corrected 4mtD3cpv response (YFP, red; CFP, blue; ratio YFP/CFP, green) in a WT fiber electrically stimulated by a single pulse in the presence of 1 mM Ca^2+^. The YFP/CFP ratio shown was obtained by using a 10-points running average. The final part of the decay phase could be well fitted to a single exponential (bright green) with a rate constant of 0.21 s^-1^.

**Comparison of the time course of the (mitochondrial) 4mtD3cpv responses and the (cytosolic) Fura-2 responses**


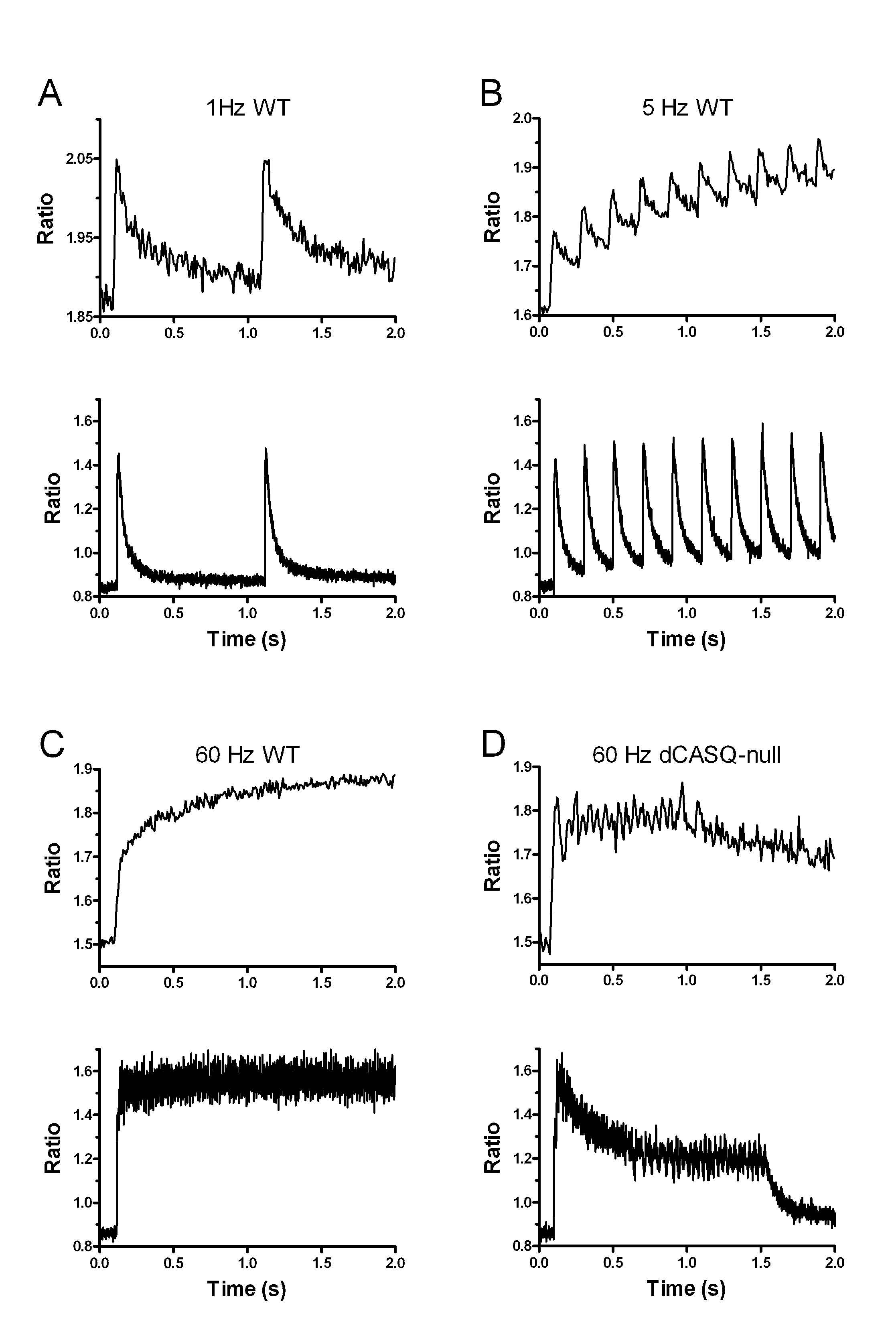


**Fig. S4. Comparison of the time course of the 4mtD3cpv responses and the Fura-2 responses.** In each panel, the upper figure shows the 4mtD3cpv response and the lower figure shows the Fura-2 response in WT at 1, 5 and 60 Hz stimulation (A, B and C) and in dCASQ-null at 60 Hz (D). Note that the decline in the 4mtD3cpv transients at 1 and 5 Hz occurs more slowly than in the Fura-2 transients. As a result there is -during the stimulation period- a gradual rise in the baseline free Ca^2+^ concentration inside the mitochondria, whereas the cytosolic Ca^2+^ concentration in between stimuli remains rather constant (Panels A and B). The comparison between WT and dCASQ-null at 60 Hz illustrates that in dCASQ-null fibers the initial peak value is similar to WT but there is a gradual decline in the cytosolic free Ca^2+^ concentration. At 60 Hz stimulation, the free [Ca^2+^]_mito_ after an early rapid increase accumulates gradually in WT but remains rather constant in dCASQ-null fibers (Panels C and D).
